# Induction of P-glycoprotein expression and activity by *Aconitum* alkaloids: Implication for clinical drug–drug interactions

**DOI:** 10.1038/srep25343

**Published:** 2016-05-03

**Authors:** Jinjun Wu, Na Lin, Fangyuan Li, Guiyu Zhang, Shugui He, Yuanfeng Zhu, Rilan Ou, Na Li, Shuqiang Liu, Lizhi Feng, Liang Liu, Zhongqiu Liu, Linlin Lu

**Affiliations:** 1 International Institute for Translational Chinese Medicine, Guangzhou University of Chinese Medicine, Guangzhou 510006, P. R. China; 2 State Key Laboratory of Quality Research in Chinese Medicine, Macau University of Science and Technology, Macau (SAR), China; 3 Institute of Chinese Meteria Medica, China Academy of Chinese Medical Sciences, Beijing 100700, P. R. China

## Abstract

The *Aconitum* species, which mainly contain bioactive *Aconitum* alkaloids, are frequently administered concomitantly with other herbal medicines or chemical drugs in clinics. The potential risk of drug–drug interactions (DDIs) arising from co-administration of *Aconitum* alkaloids and other drugs against specific targets such as P-glycoprotein (P-gp) must be evaluated. This study focused on the effects of three representative *Aconitum* alkaloids: aconitine (AC), benzoylaconine (BAC), and aconine, on the expression and activity of P-gp. We observed that *Aconitum* alkaloids increased P-gp expression in LS174T and Caco-2 cells in the order AC > BAC > aconine. Nuclear receptors were involved in the induction of P-gp. AC and BAC increased the P-gp transport activity. Strikingly, intracellular ATP levels and mitochondrial mass also increased. Furthermore, exposure to AC decreased the toxicity of vincristine and doxorubicin towards the cells. *In vivo*, AC significantly up-regulated the P-gp protein levels in the jejunum, ileum, and colon of FVB mice, and protected them against acute AC toxicity. Taken together, the findings of our *in vi*tro and *in vivo* experiments indicate that AC can induce P-gp expression, and that co-administration of AC with P-gp substrate drugs may cause DDIs. Our findings have important implications for *Aconitum* therapy in clinics.

Aconitine (AC) is one of the main bioactive *Aconitum* alkaloids present in the *Aconitum* species (*Ranunculaceae* family), and is widely used in China and other Asian countries to treat rheumatoid arthritis, cardiovascular diseases, and tumors^1^,^2^. Unfortunately, AC is also the most toxic diester alkaloid among *Aconitum* alkaloids. It can stimulate Na^+^ channels and is therefore a strong neurotoxin and painkiller^3^,^4^,^5^,^6^. For this reason, application of *Aconitum* alkaloids is restricted in clinics. When heated or hydrolyzed by the intestinal hydrolase, AC is easily converted into benzoylaconine (BAC) or aconine (Supplementary Fig. S1)^7^,^8^. Hydrolysis of AC decreases its toxicity by over 100-fold^7^,^9^,^10^.

P-glycoprotein (P-gp, MDR1) is an important protein located on the apical membrane of mature epithelial cells of different organs^11^,^12^. It functions as an efflux pump and plays a crucial role in protecting the human body by pumping external chemicals out of cells^13^. However, P-gp expression and activity are frequently changed by its own substrates, potentially affecting its pharmacokinetics, bioavailability, toxicity, and therapeutic response, which is recognized by authorities as one of the most important causes of drug-drug interactions (DDIs) among P-gp substrates^14^,^15^,^16^. Thus, investigating the effects of substrate drugs on P-gp can provide useful information for clinical use of P-gp substrate drugs.

Nuclear receptors (NRs) are ligand-inducible transcription factors that specifically regulate the expression of phase I and phase II drug-metabolizing enzymes, as well as xenobiotic transporters^16^,^17^,^18^. Among the NRs, the pregnane X receptor (PXR) and constitutive androstane receptor (CAR) are considered key transcriptional regulators of P-gp^19^. Several studies have reported the various agonists of PXR and CAR^20^,^21^,^22^,^23^,^24^,^25^, and extensive reviews have been written on the regulation of xenobiotic transporters by PXR and CAR^16^,^19^.

Previous studies have demonstrated that P-gp is the main ABC transporter involved in AC efflux^26^,^27^,^28^. Our previous studies also confirmed that P-gp mediates the transport of *Aconitum* alkaloids, and the effect of P-gp on transport follows the trend AC > BAC > aconine^29^. However, little is known about the effects of the three alkaloids on P-gp. Whether AC, BAC, or aconine can modulate P-gp via NRs, specifically via PXR or/and CAR, has never been studied. More importantly, *Aconitum* species are frequently used in combination with other herbal medicines, including *Ginseng radix*, *Zingiberis rhizoma*, *Liquiritiae radix*, *Rhei radix*, and *Paeoniae radix alba*, for toxicity reduction^30^. Some of the component herbs and ingredients have been confirmed to alter P-gp expression and activity. For example, *Liquiritiae radix* and its main bioactive compounds, including glycyrrhizin, glycyrrhetinic acid, and liquiritin, can significantly increase P-gp expression and activity^31^,^32^,^33^. Besides, *Aconitum* species are likely to be administered concomitantly with other chemical drugs that are substrates of P-gp to treat complex diseases. For example, digoxin and verapamil are substrates of P-gp, and are usually co-administered with *Aconitum* species to treat cardiovascular diseases^34^. Several anti-tumor drugs, such as paclitaxel, doxorubicin, and vincristine, are also substrates of P-gp^35^; these drugs are usually co-administrated to achieve maximum treatment efficacy against cancer. Any effect of the *Aconitum* alkaloids on P-gp expression and/or activity might cause DDIs, thereby resulting in undesirable variation in the plasma concentrations of co-administered substrate drugs, with treatment failure or toxicologically unsafe consequences. Therefore, a thorough assessment of DDI risk with co-administration of *Aconitum* alkaloids and P-gp substrates drugs is essential and urgent.

For this purpose, we first evaluated the effects of AC, BAC, and aconine on the expression of P-gp in LS174T and Caco-2 cells. These two cell lines are suitable *in vitro* models to study P-gp induction, localization, and function by xenobiotic drugs^21^,^36^,^37^,^38^,^39^. We also confirmed the regulatory effects of the tested drugs in FVB mice *in vivo*. Second, we investigated whether the three *Aconitum* alkaloids can modulate P-gp via NRs, specifically via PXR or/and CAR. Third, we explored the tested drugs on the function of P-gp in both cell lines. Fourth, we determined if changes in the expression and activity of P-gp can affect the cells against cytotoxicity produced by vincristine and doxorubicin; both drugs are used in cancer chemotherapy and as model P-gp substrates^35^. Finally, we investigated if pretreatment of AC can protect the FVB mice against acute AC toxicity. The results of this study can help predict potential risks of P-gp-related DDIs between *Aconitum* alkaloids and other co-administered drugs. Thus, our findings have important implications for the correct clinical application of *Aconitum* alkaloids.

## Results

### Effects of AC, BAC, and aconine on P-gp protein and mRNA levels

**Figure 1:**
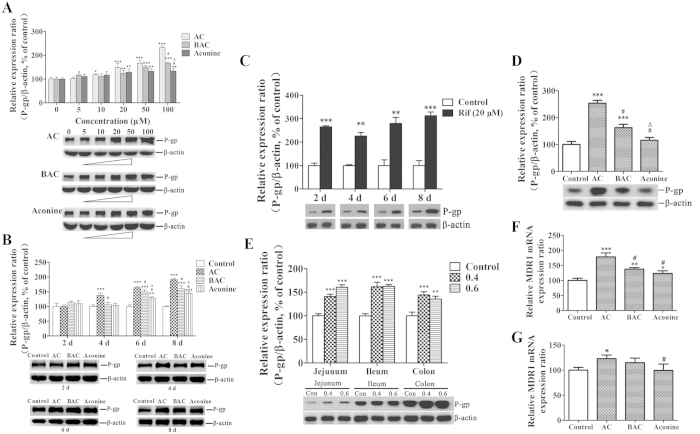
Effects of AC, BAC, and aconine on P-gp protein and mRNA levels. P-gp protein levels were detected by Western blot analysis. mRNA levels were detected by real-time PCR analysis. (**A**) Dose-dependent effects on P-gp protein levels in LS174T total lysates after treatment with the tested drugs (5–100 μM) or the vehicle (control) for 6 days. (**B**) Time-dependent effects on P-gp protein levels in LS174T total lysates after treatment with the tested drugs (50 μM) or the vehicle (control) for 2–8 days. (**C**) LS174T cells were treated with rifampicin (Rif, 20 μM) for 2–8 days as the positive control for P-gp induction. (**D**) P-gp protein levels in Caco-2 cells after treatment with the tested drugs (50 μM) or the vehicle (control) for 6 days. (**E**) P-gp protein levels in FVB mice after treatment with AC (0.4/0.6 mg/kg) or the vehicle (control) for 14 days. MDR1 mRNA levels in LS174T (**F**) and Caco-2 (**G**) cells after treatment with the tested drugs (50 μM) or the vehicle (control) for 6 days. Densitometry results were related to β-actin and presented as the percentage of controls. GAPDH was used as the housekeeping gene for cells. Data represent mean ± SD (*n* = 3). *p < 0.05, **p < 0.01, and ***p < 0.001 compared with the control group; ^#^*p* < 0.05 compared with the AC group; ^Δ^*p* < 0.05 compared with the BAC group.

**Figure 2:**
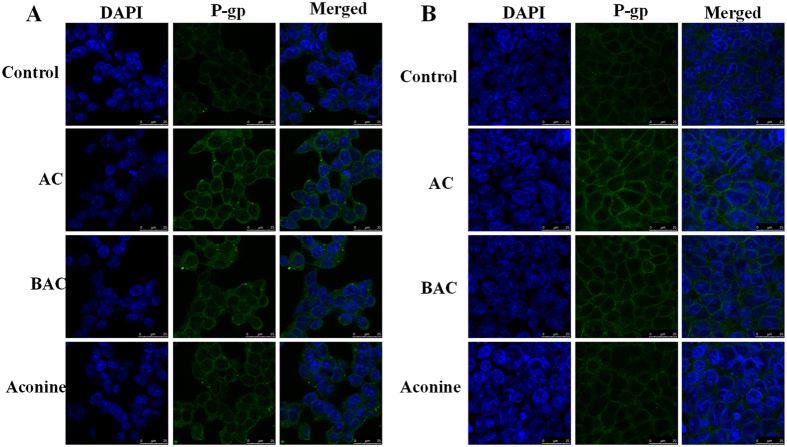
Effects of AC, BAC, and aconine on P-gp immunofluorescence in LS174T (**A**) and Caco-2 (**B**) cells by confocal microscopy. Cells were pretreated with AC, BAC, or aconine (50 μM) or with the vehicle (control) for 6 days. The scale bar represents 25 μm.

Protein levels of P-gp were evaluated by Western blot analysis. Compared with the control cells, P-gp protein levels in the tested groups gradually increased over 6 days of incubation with increasing dose in the order AC > BAC > aconine at both 50 μM and 100 μM concentration (Fig. 1A). AC also increased the P-gp protein levels in a time-dependent manner, and the increase was significantly higher than those in the BAC and aconine groups (Fig. 1B). A significant increase in P-gp levels (*p* < 0.001) was observed when rifampicin (20 μM), a model inducer of P-gp, was used to treat the LS174T cells (Fig. 1C). Treatment of Caco-2 cells with AC, BAC, and aconine (50 μM, 6 days) significantly increased the P-gp levels and the effect followed the trend AC > BAC > aconine (Fig. 1D). Treatment with 0.4 and 0.6 mg/kg AC for 14 days also significantly increased the P-gp levels in the jejunum, ileum, and colon of FVB mice (Fig. 1E). The relative MDR1 mRNA levels were further analyzed in both cell lines by real-time PCR. AC treatment (50 μM, 6 days) significantly increased the MDR1 mRNA levels in LS174T (Fig. 1F) and Caco-2 (Fig. 1G) cells in the order AC > BAC > aconine, which was consistent with our results showing increased P-gp protein levels under these conditions. As shown in Fig. 2, our immunostaining data for P-gp in LS174T and Caco-2 cells were also consistent with data obtained from Western blot analysis showing changes in total P-gp protein levels.

### NRs mediate P-gp induction via AC/BAC/aconine

**Figure 3:**
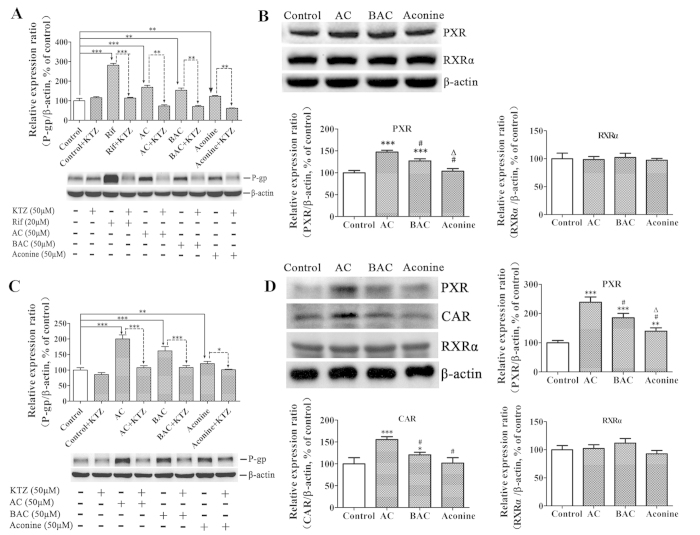
Mediation of NRs in P-gp protein up-regulation by AC, BAC, and aconine. Effects of KTZ on AC-, BAC-, or aconine-mediated P-gp induction in LS174T (**A**) and Caco-2 (**C**) cells. Ketoconazole (KTZ) (50 μM) was used as a non-specific NR inhibitor. Cells were exposed to the vehicle (control), KTZ (50 μM), AC/BAC/aconine (50 μM), or AC/BAC/aconine (50 μM) +KTZ (50 μM) for 6 days. Rifampicin (Rif, 20 μM) was used as positive control in LS174T cells. (**B**) Effects of AC, BAC, and aconine on PXR and RXRα protein levels in LS174T cells. (**D**) Effects of AC, BAC, and aconine on PXR, CAR, and RXRα protein expression levels in Caco-2 cells. Proteins were detected by Western blot analysis and presented as the percentage of control. Data represent mean ± SD (*n* = 3). *p < 0.05, **p < 0.01, and ***p < 0.001 compared with the control group or the corresponding groups; ^#^*p* < 0.05 compared with the AC group; ^Δ^*p* < 0.05 compared with the BAC group.

**Figure 4:**
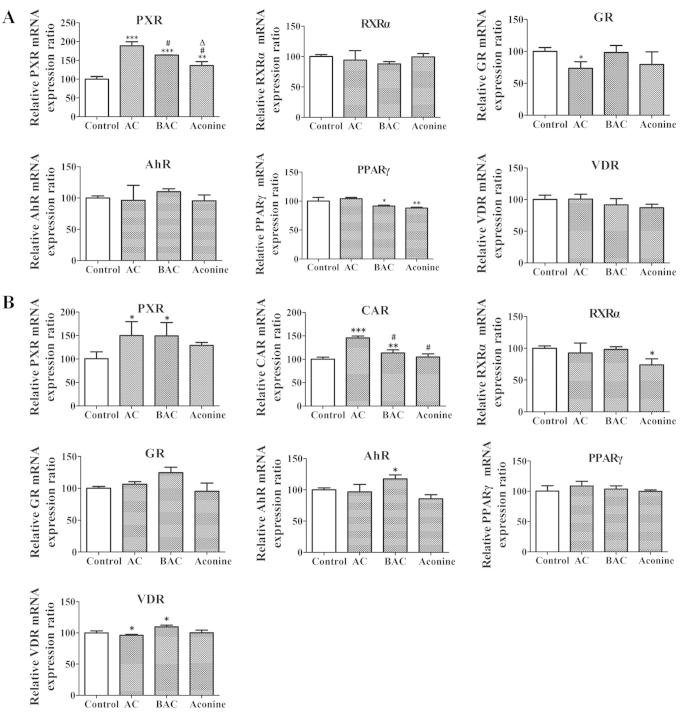
Effects of AC, BAC, and aconine on the mRNA levels of NRs. Cells were treated with AC, BAC or aconine (50 μM) or vehicle (control) for 6 days. mRNA levels were detected by real-time PCR analysis. (**A**) Effects of AC, BAC, and aconine on PXR, PPARγ, GR, AhR, VDR and RXRα mRNA expression levels in LS174T cells. (**B**) Effects of AC, BAC, and aconine on PXR, CAR, PPARγ, GR, AhR, VDR and RXRα mRNA levels in Caco-2 cells. GAPDH was used as housekeeping genes for cells. Data represent mean ± SD (*n* = 3). *p < 0.05, **p < 0.01, and ***p < 0.001 compared with the control group; ^#^*p* < 0.05 compared with the AC group; ^Δ^*p* < 0.05 compared with the BAC group.

**Figure 5:**
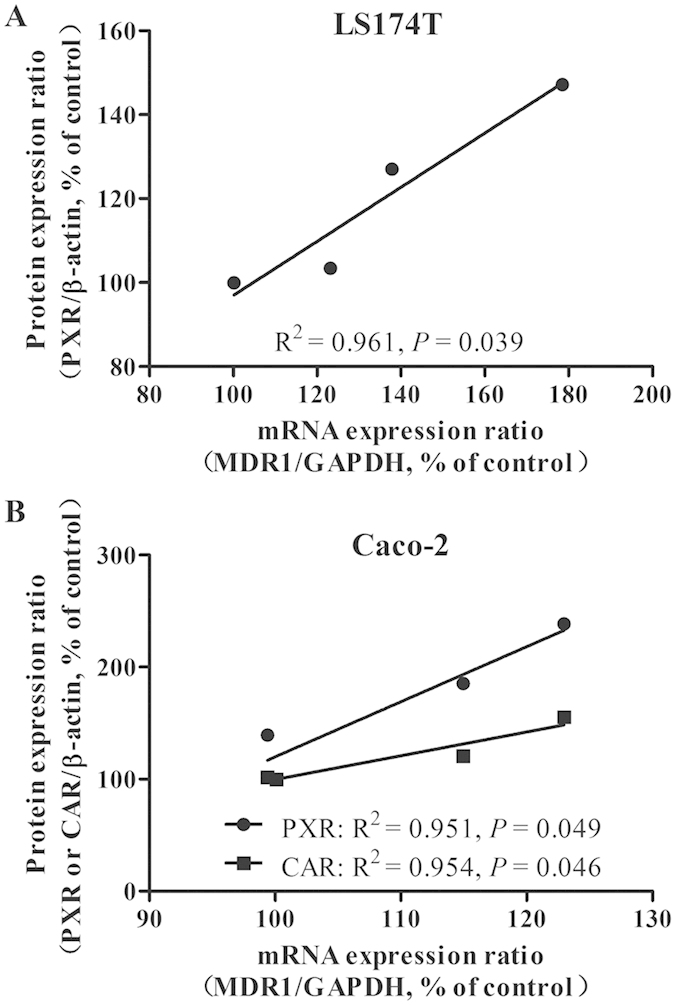
Pairwise correlation between MDR1 mRNA levels and PXR or CAR protein levels in LS174T (**A**) and Caco-2 (**B**) cells (linear regression analysis, Pearson’s correlation).

Ketoconazole (a non-specific NR inhibitor) did not modify P-gp expression in LS174T cells. However, when incorporated with AC, BAC, or aconine, ketoconazole significantly prevented increase in P-gp protein levels induced by the three alkaloids. The striking increase in P-gp levels induced by rifampicin was also significantly reversed by combined incubation with ketoconazole (Fig. 3A). The same treatment also increased PXR protein levels in LS174T cells, with the effect following the order AC > BAC > aconine. The baseline CAR levels were too low to be detected in LS174T cells in all untreated and treated groups. RXRα remained unchanged after the treatment (Fig. 3B). Ketoconazole also significantly reversed P-gp induction by the three alkaloids in Caco-2 cells (Fig. 3C). Both PXR and CAR levels increased in the order AC > BAC > aconine. No marked induction was observed in the RXRα levels (Fig. 3D). The same trend of increase in PXR mRNA levels was observed in LS174T cells (Fig. 4A). Increase in PXR and CAR mRNA levels was found in Caco-2 cells (Fig. 4B), which was consistent with protein variations. Correlations between MDR1 mRNA levels and PXR or CAR protein levels were analyzed. The MDR1 mRNA levels was closely related to the PXR protein levels in LS174T cells (*R*^2^ = 0.961, *p* < 0.05; Fig. 5A). Strong correlations were also observed between MDR1 mRNA levels and PXR protein levels (*R*^2^ = 0.951, *p* < 0.05) and CAR protein levels (*R*^2^ = 0.954, *p* < 0.05) in Caco-2 cells (Fig. 5B).

### Effects of AC, BAC, and aconine on P-gp transport activity in LS174T and Caco-2 cells

**Figure 6:**
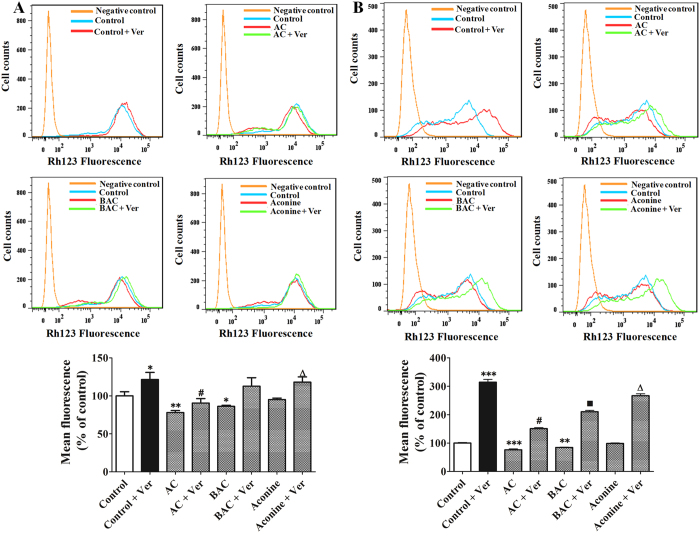
Effects of AC, BAC, and aconine on P-gp function. Cells were treated with AC, BAC, or aconine (50 μM) or with the vehicle (control) for 6 days. Rhodamine 123 (Rh123) accumulation in LS174T (**A**) and Caco-2 cells (**B**) was measured by flow cytometry, recorded in histograms in the presence or absence of verapamil (Ver) (50 μM and 100 μM for LS174T and Caco-2 cells, respectively), and inversely correlated with P-gp transport activity. Data were expressed as the percentage of control and represent mean ± SD (*n* = 3). *p < 0.05, **p < 0.01, and ***p < 0.001 compared with the control group; ^#^*p* < 0.05 compared with the AC group; ^■^*p* < 0.05 compared with the BAC group; ^Δ^*p* < 0.05 compared with the aconine group.

Intracellular accumulation of rhodamine 123 was inversely correlated with P-gp extrusion activity. Treatment of LS174T cells with AC, BAC, and aconine (50 μM, 6 days) decreased rhodamine 123 accumulation by 22% (*p* < 0.01), 14% (*p* < 0.05), and 5% (*p* > 0.05), respectively, compared to control cells (Fig. 6A). In Caco-2 cells, the same treatment also resulted in decreased rhodamine 123 accumulation by 24% (*p* < 0.001), 15% (*p* < 0.01), and 2% (*p* > 0.05), respectively (Fig. 6B). Verapamil (50 μM and 100 μM in LS174T and Caco-2 cells, respectively) was used as a positive drug for P-gp inhibition. In both cell lines, the decrease in rhodamine 123 accumulation was reversed by verapamil, which indicated that the decrease was P-gp-dependent.

### Effects of AC, BAC, and aconine on ATP levels and mitochondrial mass in LS174T and Caco-2 cells

**Figure 7:**
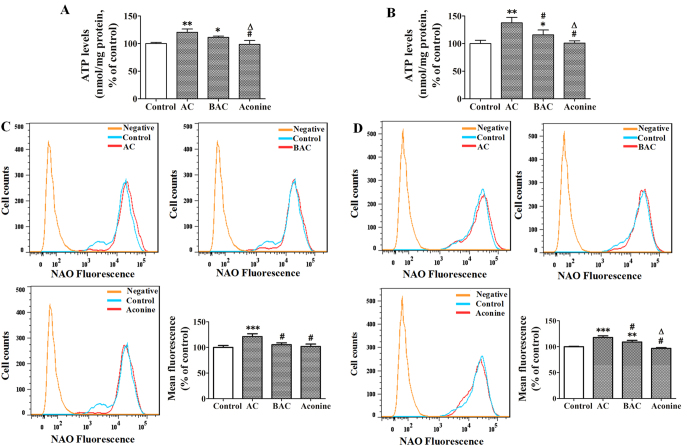
Effects of AC, BAC, and aconine on intracellular ATP levels and intracellular mitochondrial mass. Cells were treated with AC, BAC, or aconine (50 μM) or with the vehicle (control) for 6 days. ATP levels in LS174T (**A**) and Caco-2 cells (**B**) were measured with a firefly luciferase ATP assay kit. Fluorescence intensity of NAO in LS174T (**C**) and Caco-2 cells (**D**) was measured by flow cytometry and recorded in histograms. Data were expressed as the percentage of control and represent mean ± SD (*n* = 3). *p < 0.05, **p < 0.01, and ***p < 0.001 compared with the control group; ^#^*p* < 0.05 compared with the AC group; ^Δ^*p* < 0.05 compared with the BAC group.

Compared with the control, LS174T cells exposed to AC, BAC, and aconine (50 μM, 6 days) demonstrated increased intracellular ATP levels by 21% (*p* < 0.01), 11% (*p* < 0.05) and −3% (*p* > 0.05), respectively (Fig. 7A). The increase in Caco-2 cells was 38% (*p* < 0.01), 16% (*p* < 0.05), and 1% (*p* > 0.05), respectively (Fig. 7B). Incubation of LS174T cells with the same treatment increased the mean fluorescence intensity of NAO by 21% (*p* < 0.001), 5% (*p* > 0.05), and 2% (*p* > 0.05), respectively, with respect to the control cells (Fig. 7C). In Caco-2 cells, the increase in mean fluorescence intensity was 18% (*p* < 0.001), 9% (*p* < 0.01), and 2% (*p* > 0.05), respectively (Fig. 7D).

### Effects of AC, BAC, and aconine against vincristine and doxorubicin cytotoxicity

Table 1 shows the protective effect of AC/BAC/aconine (50 μM, 6 days) against vincristine and doxorubicin (two substrates of P-gp) cytotoxicity in both cell lines. In LS174T cells, the IC50 value related to vincristine cytotoxicity in the AC group was significantly higher (*p* < 0.05) than that in the control group. Similarly, incubation of Caco-2 cells with AC and BAC significantly (*p* < 0.01 and *p* < 0.05, respectively) increased the IC50 value related to vincristine cytotoxicity. Treatment of LS174T cells with AC also significantly (*p* < 0.01) increased the IC50 value related to doxorubicin cytotoxicity. Similarly, the IC50 value related to doxorubicin cytotoxicity was significantly higher (*p* < 0.01 or *p* < 0.05) in Caco-2 cells treated with AC and BAC compared to control cells. Verapamil and tariquidar were used as positive controls for P-gp inhibition. The results showed that the IC50 values related to vincristine and doxorubicin cytotoxicity were significantly lower (*p* < 0.001) in cells treated with verapamil and tariquidar compared to control cells.

### Reduction of acute AC toxicity by AC pretreatment

Table 2 shows the influence of 0.6 mg/kg of AC pretreatment for 14 days on the death of FVB mice caused by oral administration of AC (1.8 mg/kg). The number of deaths was 10 in the control group, but only 2 in the AC pretreatment group. The rate of mice death induced by administration of 1.8 mg/kg AC was significantly decreased from 52.6% to 10.5% (*p* < 0.05) after pretreatment of AC for 14 days.

## Discussion

*Aconitum* alkaloids are mainly used in China and other Asian countries to treat rheumatoid arthritis and cardiovascular diseases. However, application of *Aconitum* alkaloids is restricted because of their high toxicity. The current study is the first to explore the effects of three *Aconitum* alkaloids, namely AC, BAC, and aconine, on P-gp, and our findings are important for assessing DDIs between *Aconitum* alkaloids and other P-gp substrate drugs.

One of our major findings is that the tested drugs could significantly induce P-gp expression in both LS174T and Caco-2 cell lines. In LS174T cells, AC, BAC, and aconine induced P-gp levels in a dose- and time-dependent manner in the order AC > BAC > aconine (Fig. 1A,B), suggesting that P-gp induction by *Aconitum* alkaloids was correlated with toxicity. Based on our results and the report^21^, a concentration of 50 μM and a 6 day treatment period were selected to perform subsequent *in vitro* experiments. Rifampicin, a model inducer of P-gp^20^, induced P-gp expression as expected (Fig. 1C). Increase in P-gp levels were also detected in Caco-2 cells followed the order AC > BAC > aconine, after the same treatment (Fig. 1D). More importantly, AC significantly increased the P-gp protein levels in the jejunum, ileum, and colon of FVB mice (Fig. 1E). Next, we observed that AC significantly increased the MDR1 mRNA levels in both cell lines with consistent variation in the P-gp protein levels (Fig. 1F,G), suggesting transcriptional regulation of P-gp by the tested drugs. The immunostaining data for P-gp were consistent with Western blot data on P-gp levels (Fig. 2). Together, these data led us to conclude that AC could induce P-gp expression both *in vitro* and *in vivo*.

To evaluate whether NRs mediate the induction P-gp expression by the tested drugs *in vitro*, we performed incubations in the presence or absence of ketoconazole, an inhibitor of non-specific NR-mediated P-gp induction^40^. As shown in Fig. 3A,C, ketoconazole alone did not significantly modify P-gp levels, but markedly prevented P-gp expression from being induced by the tested drugs, thereby indicating that either of the NRs could mediate upregulation of P-gp expression. To investigate this further, we measured the protein levels of the main NRs and observed significant increases in PXR protein levels in both cell lines in the order AC > BAC > aconine (Fig. 3B,D). A similar trend of increase in the CAR protein levels was also found in Caco-2 cells (Fig. 3D). We could not determine CAR protein levels in the treated LS174T cell line because of its low baseline CAR levels^37^,^41^. The ligand RXRα remained unchanged. An additional study was conducted to determine the mRNA levels of the main NRs. Only a significant increase in PXR and CAR mRNA levels was observed followed the order AC > BAC > aconine (Fig. 4). Strong positive correlations were found between MDR1 mRNA induction and increase in PXR and CAR protein levels (Fig. 5), suggesting that the tested drugs induced P-gp, likely via activation of the PXR and CAR pathways.

Another major finding of this study was the functional consequences of P-gp induction *in vitro*. P-gp efflux activity was evaluated by the ability of the cells to carry out rhodamine 123, a typical P-gp substrate^23^. As shown in Fig. 6A, AC and BAC significantly increased P-gp activity in LS174T cells, which were reflected in the decreased intracellular content of rhodamine 123. Obviously, increase in P-gp activity followed the trend AC > BAC > aconine, which indicated that changes in P-gp function corresponded to changes in P-gp expression. A similar response was also observed in Caco-2 cells (Fig. 6B). Verapamil, a classical P-gp-mediated drug transport inhibitor, could reverse the decrease in rhodamine 123 accumulation in both cell lines, suggesting that the decrease was P-gp-dependent.

P-gp is an efflux pump and its activity is directly dependent on energy released by ATP hydrolysis. Thus, measurement of intracellular ATP levels is a commonly used approach to explore the mechanism of changes in P-gp function^42^. In both cell lines, AC and BAC significantly increased intracellular ATP levels (Fig. 7A,B). Cardiolipin is an important parameter for investigating intracellular events and a critical indicator of mitochondrial function^43^. The role of cardiolipin in biological membranes is to accomplish oxidative phosphorylation leading to ATP synthesis^44^,^45^. Thus, we further measured the quality of mitochondria in cells using NAO, which acts as a highly specific probe for cardiolipin^43^. As shown in Fig. 7C,D, the mean fluorescence intensity of NAO in cells exposed to AC significantly increased, which indicated that AC could increase mitochondrial mass. However, this response was modest or absent in the BAC or aconine groups. Taken together, these results suggest that increased intracellular ATP levels and mitochondrial mass may mediate the increase in P-gp function induced by AC and BAC.

Finally, induction of P-gp has been shown to confer resistance to a vast array of chemotherapeutic drugs because of its ability to increase drug efflux^46^,^47^. We postulated that AC could decrease the toxicity produced by chemotherapeutic agents towards cells because of its induction of P-gp. Our results showed that AC significantly increased the IC50 values related to vincristine and doxorubicin cytotoxicity relative to control cells (Table 1), which indicated that AC decreased the toxicity of the tested anticancer drugs towards the cells. In addition, pretreatment of AC could significantly reduce the rate of death of FVB mice caused by oral administration of AC (Table 2). Given these results, we speculate that AC pretreatment may protect mice against AC acute toxicity through P-gp induction in the jejunum, ileum, and colon of FVB mice, thereby decreasing AC absorption. However, it is also possible that AC can induce CYP3A4 or/and CYP2D6, which are the most important CYP isoforms responsible for AC metabolism^48^, thereby accelerating AC metabolism.

In summary, we have demonstrated increased P-gp expression in response to treatment with three *Aconitum* alkaloids *in vitro* and *in vivo*. AC produced stronger induction of P-gp than BAC and aconine. Furthermore, P-gp induction was mediated in part via NRs, and PXR and CAR are likely to be part of this mechanism. AC also activated P-gp transport activity in LS174T and Caco-2 cells, concomitantly decreasing the toxicity of vincristine and doxorubicin towards cells. In addition, AC protected mice against acute AC toxicity. We identified possible DDIs of P-gp when *Aconitum* alkaloids, especially AC, are co-administered with other P-gp substrates. Clinical use of *Aconitum* alkaloid-related therapy should be carefully monitored to avoid adverse interactions.

## Materials and Methods

### Materials

AC, BAC, and aconine (purity >98%) were purchased from Chengdu Mansite Pharmaceutical Co., Ltd. (Chengdu, China). Vincristine, doxorubicin and tariquidar (purity >98%) were purchased from Dalian Meilun Biological Technology Co., Ltd. (Dalian, China). Rifampicin, verapamil, rhodamine 123, dimethyl sulfoxide (DMSO), ketoconazole, phenylmethylsulfonyl fluoride, 3-(4,5-dimethyl-2-thiazolyl)-2,5-diphenyl-2-H-tetrazolium bromide (MTT) and nonyl acridine orange (NAO) were purchased from Sigma–Aldrich (St. Louis, MO, USA). RIPA lysis buffer, BCA protein assay kit, and ATP level detection kit were purchased from Beyotime Institute of Biotechnology (Haimen, Jiangsu, China). All other chemicals and solvents were of analytical grade or better and used as received.

## Experiments In Human Cell Lines

### Cell lines and cell culture

The human LS174T cell line was purchased from Shanghai Type Culture Collection of Chinese Academy of Sciences (Shanghai, China). The human Caco-2 cell line was provided by Dr. Ming Hu (Department of Pharmacological and Pharmaceutical Sciences, College of Pharmacy, University of Houston, Houston, TX, USA). LS174T and Caco-2 cells were cultured in DMEM containing 10% (v/v) fetal bovine serum, 100 U/ml penicillin, and 100 μg/ml streptomycin. The cells were grown at 37 °C in a humidified atmosphere with 5% CO_2_.

### Cell treatments

To investigate the time-dependent effects on P-gp expression in LS174T, cells were seeded in six-well plates and incubated with AC, BAC, aconine (50 μM), or the DMSO vehicle control for 2, 4, 6, and 8 days. Cells were seeded at different densities (8 × 10^5^, 5 × 10^5^, 3 × 10^5^, and 1 × 10^5^ cells/well for 2, 4, 6, and 8 days, respectively) to avoid over-confluence. To assess the dose-dependent effects on P-gp expression following treatment with the tested drugs, cells were seeded at 3 × 10^5^ cells/well and incubated with AC, BAC, or aconine (5, 10, 20, 50, and 100 μM) for 6 days. As positive control, LS174T cells were exposed to rifampicin (20 μM) for P-gp induction. Induction experiment was also conducted in Caco-2 cells. Caco-2 cells were seeded at 3 × 10^5^ cells/well and incubated with AC, BAC, aconine (50 μM), or the DMSO vehicle control for 6 days. The medium was renewed on alternative days when a fresh drug or vehicle was added.

Ketoconazole was used to explore the mediation effects of NRs on P-gp expression induced by AC, BAC, and aconine. Cells were seeded at 3 × 10^5^ cells/well in six-well plates and exposed to the vehicle (DMSO, control), ketoconazole (50 μM), AC/BAC/aconine (50 μM), or AC/BAC/aconine (50 μM) + ketoconazole (50 μM) for 6 days.

The cytotoxicity of AC, BAC, and aconine on LS174T and Caco-2 cells were evaluated using the MTT assay. Both cell lines were cultured in the 96-well plates for 24 h at a seeding density of 1.0 × 10^4^ cells/well before the addition of drugs. After treatment with AC, BAC, and aconine at different concentrations (0, 1, 10, 50, 100, 200, or 400 μM) for different times (24, 48, 72, or 96 h), the medium was removed and 100 μl of MTT reagent (0.5 mg/ml) was added to each well for another 4 h of incubation. At the end of the incubation period, the medium was removed; intracellular formazan was solubilized with 150 μl of DMSO and quantified spectrophotometrically (λ = 570 nm) with a microplate reader Victor X3 (PerkinElmer, Waltham, MA, USA). The percentage of cell viability was calculated based on the measured absorbance relative to the absorbance of control cells. The rate of MTT conversion in all the treated groups was not statistically different from the respective control cells (Supplementary Fig. S2).

### Western blot analysis

Cell lysates were prepared with RIPA lysis buffer containing 1 mM phenylmethylsulfonyl fluoride as a protease inhibitor. Protein concentrations were determined with a BCA estimation kit according to the manufacturer’s instructions. Cell protein (40 μg) was loaded onto each lane and separated by SDS-PAGE (4% stacking gel; 10% separating gel). The separated proteins were transferred from the gel to the PVDF membrane. After blocking for 1 h with non-fat milk (5%, w/v) in Tris-buffered saline containing 0.1% Tween-20 (TBST), the primary antibody of P-gp (Abcam, Cambridge, UK), PXR, CAR, RXRα, or β-actin (Santa Cruz Biotechnology, Santa Cruz, CA, USA) at a dilution of 1:1000 was added to TBST with 5% non-fat milk and then incubated with the membrane at 4 °C overnight. The membrane was washed before incubation with the corresponding secondary antibody at a dilution of 1:3000 in the same buffer for 1 h at room temperature. Western blot signals were detected with an ECL chemiluminescence detection agent. The relative intensity of each protein band was scanned and quantified by Quantity One (Bio-Rad, Hercules, CA, USA).

### Confocal microscopy

Both LS174T and Caco-2 cells were seeded on sterile glass cover slips in six-well plates. Cells were treated with AC, BAC, or aconine at the final concentration of 50 μM for 6 days. At the end of the treatment, cells were washed with PBS three times and fixed in 4% paraformaldehyde for 30 min. After three washes with PBS, cells were permeabilized with 0.5% Triton X-100 for 20 min and washed with PBS for another three times. Once cells were fixed and permeabilized, they were blocked with 5% bovine serum albumin for 1 h at room temperature before treatment with monoclonal anti-P-gp antibody (1:100; F4 clone, Sigma–Aldrich, St. Louis, MO, USA) at 4 °C overnight. The cells were washed with PBS and stained with secondary fluorescent antibody (1:200; Alexa Fluor 488, Santa Cruz Biotechnology, Santa Cruz, CA, USA) for 1 h at room temperature. Subsequently, cells were washed with PBS and incubated with DAPI (5 μg/ml) for another 20 min. Fluorescent signals were detected with a confocal fluorescence microscope (Leica, Germany).

### Real-time PCR analysis

After cell treatment of AC, BAC, or aconine (50 μM, 6 days), total RNA was extracted by the TRIzol extraction method (Invitrogen, Carlsbad, CA, USA) according to the manufacturer’s instructions. cDNA was reverse-transcribed from total RNA with a reverse transcription kit (TaKaRa, Shiga, Japan). SYBR Green real-time PCR amplification and detection were performed using an ABI 7500 fast system (Applied Biosystems, Foster City, CA, USA). The sequences of the primers are listed in Supplementary Table S1. The thermal profile for real-time PCR was 95 °C for 30 s, 95 °C for 5 s, and 60 °C for 34 s. A melting curve was also obtained. Target mRNA levels were normalized against GAPDH mRNA levels, and all samples were run in triplicate.

### Transport activity of P-gp

The efflux activity of P-gp was evaluated by the ability of the cells to detect the fluorescent compound rhodamine 123. Cells were seeded at 3 × 10^5^ cells/well in six-well plates. After cell treatment of AC, BAC, aconine (50 μM, 6 days) or the DMSO vehicle control, the medium was replaced with fresh medium containing Rh123 (final concentration, 1 μM) with or without verapamil (50 μM and 100 μM for LS174T cells and Caco-2 cells, respectively). After 90 min of incubation at 37 °C, the cells were washed twice with cold PBS and re-suspended in PBS for further measurement. Cells that had not been exposed to rhodamine 123 were used as negative controls. The fluorescence intensity of rhodamine 123 was measured by a BD FACSAria III flow cytometer (BD Biosciences, San Jose, CA), with a 488 nm argon laser and FL1 channel. All the data were analyzed by Flow Jo 7.6.1 software (Tree Star, Inc., Ashland, OR, USA). The mean fluorescence value was converted to the percentage of control.

### ATP determination

Cellular ATP levels were measured using a firefly luciferase ATP assay kit according to the manufacturer’s instructions. In brief, cells were seeded at 3 × 10^5^ cells/well in six-well plates. After cell treatment of AC, BAC, aconine (50 μM, 6 days) or the DMSO vehicle control, the cells were washed twice with cold PBS and lysed with the ATP-releasing reagent provided by the kit. The luciferin substrate and luciferase enzyme were added, and bioluminescence was assessed by a Victor X3 spectrofluorometer (PerkinElmer, Waltham, MA, USA). Standard curves were also generated, and the protein concentration of each treatment group was determined with the BCA protein assay kit. The intracellular ATP level was normalized by the protein content in each sample (nmol/mg protein). The level of cellular ATP was converted to the percentage of control.

### Detection of mitochondrial mass

Cells were seeded at 3 × 10^5^ cells/well in six-well plates. After cell treatment of AC, BAC, aconine (50 μM, 6 days) or the DMSO vehicle control, the medium was replaced with fresh medium containing NAO (final concentration, 500 nM). After 30 min of incubation at 37 °C, the cells were washed twice with cold PBS and re-suspended in PBS for measurement. The mean fluorescence intensity of NAO was measured by a BD FACSAria III flow cytometer (BD Biosciences, San Jose, CA, USA), with a 488 nm argon laser and FL1 channel. All the data were analyzed using Flow Jo 7.6.1 software (Tree Star, Inc., Ashland, OR, USA). The mean fluorescence value was converted to the percentage of control.

### Cytotoxicity assay

The MTT colorimetric assay was used to test the potential role of AC, BAC, or aconine against the cytotoxicity produced by the antitumor drugs vincristine and doxorubicin. Cells were seeded in 96-well plates at a density of 8 × 10^3^ cells/well. After incubation at 37 °C for 24 h, cells were pretreated with AC, BAC, aconine (50 μM, 6 days) or the DMSO vehicle control and then incubated with fresh medium containing different concentrations of vincristine (0–800 μM) or doxorubicin (0–200 μM) in the presence or absence of verapamil (50 μM for LS174T cells and 100 μM for Caco-2 cells), or tariquidar (500 nM) for an additional 48 and 96 h for LS174T and Caco-2 cells, respectively. Cell viability was measured by the MTT assay, as described above. The IC50 values, which represent the concentration of vincristine or doxorubicin resulting in 50% viability, were calculated using GraphPad Prism 5.0 (GraphPad Software, La Jolla, CA, USA).

## Experiments in Mice

### Animals and treatments

Male FVB mice (20–22 g) were supplied by Vital River Laboratory Animal Technology Co., Ltd. (Beijing, China; license: SCXK, Beijing, 2012-0001). The mice were kept in an environmentally controlled room (temperature: 23 °C–25 °C; relative humidity: 40%–70%; 12 h light/dark cycle) with free access to standard feed and water. The mice were randomly divided into three groups. The AC group orally received AC at 0.4 mg/kg (*n* = 8) and 0.6 mg/kg (*n* = 7) once per day over 14 consecutive days. The control group (*n* = 6) received only the same volume of the vehicle (0.1% DMSO) for the same time period. The animal experiments were performed in accordance with the care and use of laboratory animals and were approved by the ethics committee of Guangzhou University of Chinese Medicine (Guangzhou, China).

### Specimen collection

All the mice were sacrificed by cervical dislocation after administration of AC for 14 days. The jejunum, ileum and colon were removed. The total tissue proteins were obtained by scraping and homogenized with RIPA lysis buffer containing 1 mM phenylmethylsulfonyl fluoride as a protease inhibitor. The protein concentrations were determined with the BCA estimation kit according to the manufacturer’s instructions.

### Western blot analysis

The proteins in the jejunum, ileum or colon (60 μg) from different pretreatment groups were separated by SDS-PAGE. Other detailed procedures were described above. The primary antibodies of P-gp (Abcam, Cambridge, UK) or β-actin (Santa Cruz Biotechnology, Santa Cruz, CA, USA) were used.

### Determination of mortality in mice given AC after AC induction for 14 days

Male FVB mice (20–22 g) were randomly divided into two groups with 19 mice in each group. The mice were kept in the same environmentally controlled room described above. The AC group orally received AC at 0.6 mg/kg once per day over 14 consecutive days. The control group received only the same volume of the vehicle (0.1% DMSO) for the same time period. On the fifteenth day, each group of mice was orally given 1.8 mg/kg of AC. Mortality and signs of acute toxicity were monitored and recorded during a 24-period after the administration of AC. Death rate were calculated by dividing the number of death animals with the total number of animals in each group.

### Statistical analysis

All the assays were performed in three independent experiments. All results were presented as mean ± standard deviation (SD). Significant differences were analyzed using Student’s *t*-test (two groups) or one-way ANOVA followed by the LSD post-hoc test (for more than two groups) by SPSS 19.0. Significance of death rate was analyzed using χ^2^ test, and correlation analyses were performed using Pearson product–moment correlation by SPSS 19.0. Statistical differences were considered significant at *p* < 0.05.

## Additional Information

**How to cite this article**: Wu, J. *et al.* Induction of P-glycoprotein expression and activity by *Aconitum* alkaloids: Implication for clinical drug–drug interactions. *Sci. Rep.* **6**, 25343; doi: 10.1038/srep25343 (2016).

## Supplementary Material

Supplementary Information

## Figures and Tables

**Table 1:** Effects of AC, BAC, and aconine on vincristine and doxorubicin-induced cytotoxicity.

Treatment	LS174T IC_50_ (μM)	Caco-2 IC_50_ (μM)
Vincristine (Control)	56.81 ± 3.67	356.10 ± 7.05
+AC	71.83 ± 4.20*	443.47 ± 18.17**
+BAC	63.95 ± 6.75	403.60 ± 34.30*
+Aconine	57.59 ± 8.28^#^	382.33 ± 29.60^#^
+Ver	2.40 ± 0.48***	95.62 ± 10.12***
+Tar	7.44 ± 1.29***	67.01 ± 2.20***
Doxorubicin (Control)	5.86 ± 0.44	94.86 ± 4.02
+AC	8.93 ± 1.86**	112.60 ± 4.47**
+BAC	6.10 ± 0.30^#^	105.97 ± 3.16*
+Aconine	5.58 ± 0.97^#^	100.46 ± 4.41^#^
+Ver	0.54 ± 0.03***	1.07 ± 0.09***
+Tar	0.0033 ± 0.00035***	0.51 ± 0.11***

Cell viability was measured by the MTT assay. Data were expressed as the percentage of control and represent mean ± SD (*n* = 3). Verapamil (Ver) and tariquidar (Tar) were used as positive controls of P-gp inhibitor. *p < 0.05, **p < 0.01, and ***p < 0.001 compared with the control group; ^#^*p* < 0.05 compared with the AC group.

**Table 2:** Influence of 0.6 mg/kg of AC pretreatment for 14 days on the death rate of FVB mice induced by oral administration of AC (1.8 mg/kg).

Group	Pretreatment for 14 days	Dosage of AC (mg/kg)	Number of animal	Number of death	Death rate (%)	*p* value
1	0.1% DMSO	1.8	19	10	52.6	–
2	0.6 mg/kg of AC	1.8	19	2	10.5	*p* < 0.05
