# Supplementary material for: Induction of P-glycoprotein expression and activity by Aconitum alkaloids: Implication for clinical drug–drug interactions
Source: Sci Rep. 2016 May 3;6:25343. doi: 10.1038/srep25343 (PMC4853792; doi:10.1038/srep25343)
Supplement: Supplementary Information [file srep25343-s1.pdf]

Supplementary Information

Induction of P-glycoprotein expression and activity by *Aconitum* alkaloids: Implication for clinical drug–drug interactions

Jinjun Wu[a], Na Lin[a, c,], Fangyuan Li[a], Guiyu Zhang[a], Shugui He[a], Yuanfeng Zhu[a], Rilan Ou[a], Na Li[b], Shuqiang Liu[a], Lizhi Feng[a], Liang Liu[b], Zhongqiu Liu[a, b], Linlin Lu [a, b, *]

[a] International Institute for Translational Chinese Medicine, Guangzhou University of Chinese Medicine, Guangzhou 510006, PR China

[b] State Key Laboratory of Quality Research in Chinese Medicine, Macau University of Science and Technology, Macau (SAR), China

[c] Institute of Chinese Meteria Medica, China Academy of Chinese Medical Sciences, Beijing 100700, PR China

* Corresponding author:

Linlin Lu, International Institute for Translational Chinese Medicine, Guangzhou University of Chinese Medicine, Guangzhou 510006, PR China. E-mail: lllu@gzucm.edu.cn. Tel: +8620-39357902. Fax: +8620-39358071.

Supplementary Table S1

Gene-specific polymerase chain reaction primers

Gene	Forward primer (5' to 3')	Reverse primer (5' to 3')	Size (bp)
**MDR1 (NM_000927)**	TGCTCAGACAGGATGTGAGTTG	AATTACAGCAAGCCTGGAACC	122
**PXR (NM_003889)**	AAATCTGCCGTGTATGTGGGG	GGGTCTTCCGGGTGATCTC	156
**CAR (NM_001077482)**	GTGCTTAGATGCTGGCATGAGGAA	GGCTGGTGATGGATGAACAGATGAG	231
**RXRα (NM_002957)**	CATCTTTGACAGGGTGCTGA	GGGTTAAAGAGGACGATGGC	102
**GR (AB307716)**	AACTCTGCCTGGTGTGCTCTGA	TGTGCTGTCCTTCCACTGCTCT	105
**PPARγ (AB307692)**	AAGGAGAAGCTGTTGGCGGAGA	CAGCCCTGAAAGATGCGGATGG	100
**AhR (NM_001621)**	GATGCTTTGGTCTTTTATGC	TTCCCTTTCTTTTTCTGTCC	368
**VDR (AF026260)**	AGGCGAAGCATGAAGCGGAAG	ATGGTGGGCGTCCAGCAGTAT	276
**GAPDH (NM_001256799)**	GGCCTCCAAGGAGTAAGACC	AGGGGAGATTCAGTGTGGTG	122

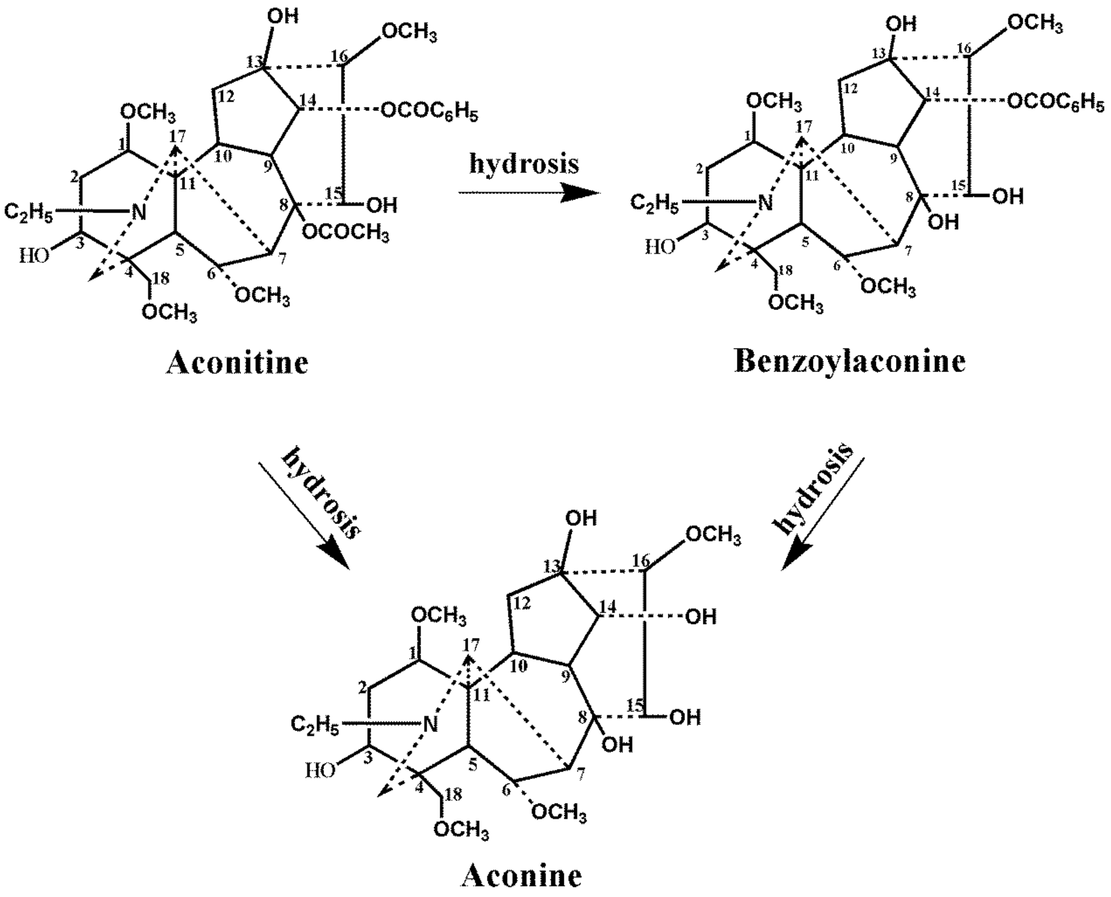

Supplementary Figure S1. Chemical structures of aconitine (AC), benzoylaconitine (BAC) and aconine.

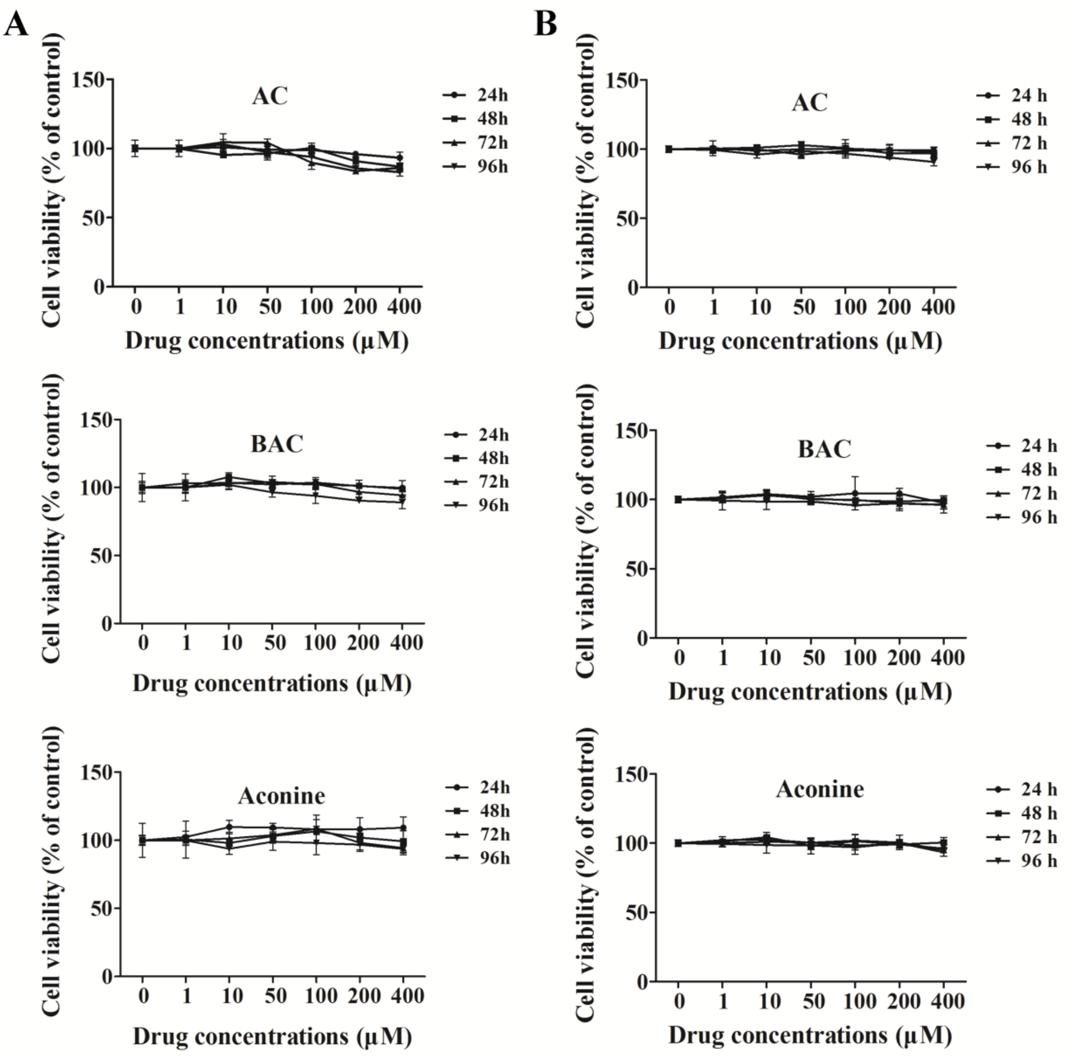

Supplementary Figure S2. Effects of AC, BAC, and aconine on LS174T (A) and Caco-2 (B) cells viability using MTT assay. The cells were exposed to AC, BAC, and aconine at different concentrations (0–400 μM) or solvent (control cells) for 24, 48, 72, and 96 h. The data (% viability) were expressed as mean ± SD (n = 6).
